# Lightweight Visual Detection and Dynamic Tracking for Pigeon Egg Inspection in Caged Pigeon Farming

**DOI:** 10.3390/s26113283

**Published:** 2026-05-22

**Authors:** Qianhui Li, Yufan Cheng, Jingcheng Xi, Zhenghang He, Qingqing Ye, Chang Zhu, Rui Kang, Longshen Liu

**Affiliations:** College of Smart Agriculture (College of Artificial Intelligence), Nanjing Agricultural University, Nanjing 211800, China; liqianhui@stu.njau.edu.cn (Q.L.); yeqingqing@stu.njau.edu.cn (Q.Y.);

**Keywords:** pigeon egg detection, quality assessment, tracking algorithm, smart farming, lightweight model

## Abstract

Manual inspection in large-scale pigeon farms is inefficient and often misses critical targets. In addition, recognition results are difficult to link to physical cage locations in real time. Here, we develop an intelligent inspection and localization system that integrates an improved lightweight YOLO model with QR-code-based tracking. QR codes are deployed along the inspection route as spatial anchors. Base detection models are combined with the ByteTrack algorithm to establish a dynamic mapping among video frames, cage numbers and detected targets. To improve the detection of small pigeon eggs caused by interference from metal cage meshes, we further design a lightweight YOLO-PEDI (Pigeon Egg Detection Inspection) model. Ghost modules replace standard convolutions to reduce computational cost. CBAM is introduced to enhance feature extraction in complex backgrounds. The newly designed model enables simultaneous identification of egg number and egg condition, including normal and broken eggs. The proposed method achieves an mAP50 of 98.1%, with only 1.53 million parameters and an inference time of 0.8 ms. Field tests show a cumulative egg-counting accuracy of 80.0% and a broken egg detection rate of 98.0%. These results demonstrate the potential of the proposed system for intelligent inspection in pigeon farming and provide a practical route towards precise traceability and digital production management.

## 1. Introduction

In recent years, changing consumer preferences and growing demand for high-protein functional foods have increased interest in pigeon eggs [1]. Pigeon eggs are valued for their high-quality protein, lecithin and trace elements [2]. Compared with conventional poultry eggs, they are smaller and have more fragile shells [3]. In multi-layer cage farming systems, egg counting and quality assessment still rely mainly on manual inspection [4]. This approach is labor-intensive, inefficient and prone to causing shell breakage during handling, which may result in economic losses in large-scale production. In addition, manual recording cannot effectively associate inspection results with physical cage locations in real time. This weakens production traceability and limits the digital development of smart pigeon farms.

Deep-learning-based object detection has advanced rapidly in intelligent agricultural monitoring [5]. Among existing methods, the YOLO (You Only Look Once) series has shown strong real-time performance and good deployment flexibility in tasks such as fruit and vegetable counting, livestock and poultry monitoring, and crop pest and disease detection [6]. These advantages arise from its end-to-end architecture and fast inference speed. With the growing demand for on-site deployment, lightweight detection models have become an important research direction, especially for embedded systems with limited computing resources [7]. Despite these advances, direct application of existing object detection methods to pigeon egg inspection remains challenging. Pigeon eggs are small targets with low pixel occupancy, making their features vulnerable to loss during down-sampling and multi-scale feature extraction [8]. In addition, metal cage meshes, pigeon feathers and complex backgrounds often interfere with target recognition [9]. Mobile inspection platforms are also usually equipped with embedded edge devices with limited computing power and storage, which restrict the real-time deployment of large detection models [10]. To address these computational constraints, researchers have explored various lightweight architectures. MobileNet utilizes depth-wise separable convolutions to reduce FLOPs, while ShuffleNet employs channel shuffling to maintain cross-channel information flow [11]. Compared with these alternatives, the Ghost module provides a suitable lightweight strategy for agricultural inspection by generating additional “cheap” feature maps through linear transformations. Although depth-wise separable convolution can effectively reduce parameter count and computational cost, its channel-wise independent operation may weaken the feature redundancy required to identify subtle biological contours, especially when small pigeon eggs are embedded in high-frequency structural noise caused by galvanized meshes. In contrast, the Ghost module reduces redundant computation while preserving a certain degree of intrinsic feature redundancy, thereby providing a more favorable accuracy–latency trade-off for small-target detection in complex poultry-house environments [12].

Researchers have proposed various strategies to improve small-target detection and enable lightweight deployment in resource-limited environments. Among them, the Ghost module has attracted considerable attention because it can reduce redundant feature generation and significantly lower computational cost while maintaining feature representation capability [13]. This advantage makes it particularly suitable for embedded vision systems that require fast inference and low memory consumption. In parallel, attention mechanisms have been widely introduced to strengthen the extraction of discriminative features. The convolutional block attention module (CBAM) [14], for example, enhances feature learning by adaptively refining channel and spatial responses, allowing the network to focus more effectively on subtle target regions and suppress background interference. This selective attention is particularly critical for pigeon egg detection, where high-frequency noise from galvanized metal meshes often leads to spurious activations in baseline architectures. By sequentially refining channel and spatial responses, CBAM facilitates a strategic trade-off between representational capacity and model size, ensuring that the model prioritizes discriminative biological features over structural farm artifacts. These strategies have achieved promising results in agricultural applications such as fruit counting, seed classification and crop disease detection. However, their use in fragile egg inspection remains limited, especially in tasks that require both accurate detection and real-time association with physical cage locations [15].

In practical pigeon farming, egg inspection involves not only target detection but also continuous tracking, cage-level localization, and production data recording. This makes the task more complex than conventional object detection. A useful inspection system must identify small and fragile eggs under occlusion, operate efficiently on mobile embedded platforms, and establish a stable connection between visual results and cage units. Existing studies have mainly focused on improving detection accuracy, while less attention has been given to integrating lightweight perception, dynamic tracking and spatial traceability into a unified framework. As a result, current approaches still have limited applicability in real breeding environments, where real-time inspection, production statistics and digital management are all required. In the domain of agricultural traceability, multi-target tracking (MTT) algorithms are essential for maintaining identity continuity. While Simple Online and Realtime Tracking (SORT) focuses primarily on motion cues [16], ByteTrack improves stability by associating low-score detection boxes, which significantly reduces identity fragmentation during intermittent occlusions by breeding pigeons. Integrating such tracking algorithms with QR-code spatial anchors offers a robust pathway for establishing a “one cage, one record” paradigm.

To address these limitations, this study proposes an improved YOLO-PEDI model for dynamic pigeon egg inspection. The Ghost module [17] is introduced into the backbone to reduce parameter size and floating-point operations (FLOPs) [18], thereby improving real-time inference performance on embedded platforms. CBAM [19] is further incorporated to enhance sensitivity to fine-grained features, such as cracked and deformed eggs, while preserving lightweight deployment. In addition to model improvement, the proposed mobile inspection platform integrates ByteTrack for multi-target tracking, continuous counting and status updating during movement [20,21,22]. QR codes are deployed on cage units to associate visual inspection results with physical locations, enabling spatial binding and traceability of inspection data. Together, these components establish a closed-loop framework for detection, counting, grading, positioning and data feedback. The proposed system enables real-time recording and analysis of egg quantity and quality at the individual cage level, and provides technical support for breeding performance evaluation, production traceability and digital management in pigeon farms.

In summary, this paper develops a lightweight, integrated system for visual inspection and spatial traceability tailored to the requirements of factory inspection for pigeon eggs. The main contributions include: (1) proposing YOLO-PEDI, a small-target lightweight detection model designed for complex cage environments, to improve the accuracy of pigeon egg counting in video streams; (2) integrating the Ghost module with the CBAM attention mechanism to reduce computational overhead while enhancing the recognition of fine-grained defects; (3) introducing an innovative tracking QR code cascading spatial positioning mechanism for integrated closed-loop monitoring of counting, grading, and cage association.

## 2. Materials and Methods

### 2.1. Data Acquisition and Preprocessing

**Figure 1:**
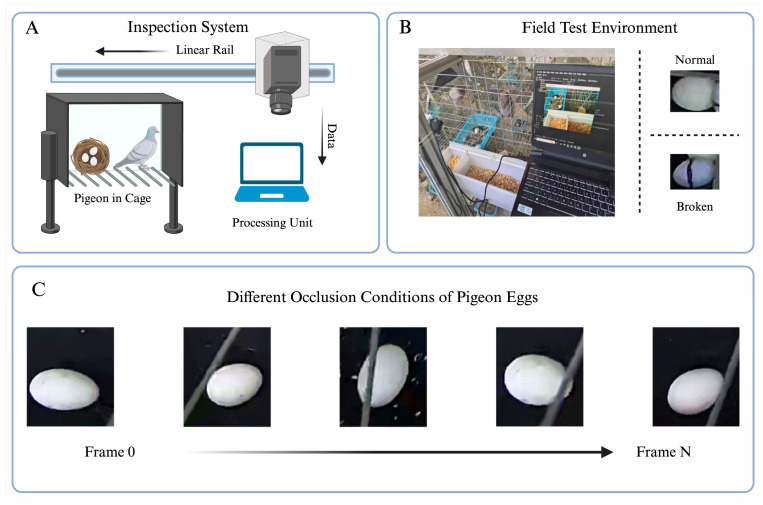
Rail-based intelligent inspection system for pigeon eggs. (**A**) The rail-mounted intelligent inspection robot and its deployment in the pigeon house; (**B**) Re-al-time computer vision processing pipeline for video streams; (**C**) Example of multi-frame sam-pling under metal mesh occlusion conditions.

The experimental data used in this study were collected from October to December 2025 at Nanjing Dongchen Pigeon Industry Co., Ltd., located in Tuanjie Community, Longpao Sub-district, Liuhe District, Nanjing City, Jiangsu, China. The experiments were conducted in a standard three-tier cage pigeon house. The cages were constructed from galvanized metal mesh, and indoor illumination was regulated by an automated supplemental lighting system. As shown in Figure 1A, image acquisition was performed using a self-developed rail-mounted intelligent inspection robot. The robot was suspended above the cage array on an I-shaped aluminum-alloy rail and was equipped with an automatic feeding module at its base, allowing visual inspection during feeding. The visual sensing unit consisted of industrial high-definition cameras mounted on the top of the robot through adjustable brackets. The optical axis was oriented approximately 35° downward from the horizontal plane, ensuring that the field of view fully covered both the pigeon eggs inside the nest and the QR-code label located at the edge of the cage opening. During operation, the robot was driven by a stepper motor and moved linearly along the rail at a constant speed of 0.2–0.5 m/s. As shown in Figure 1B, the video stream was processed in real time using computer vision. As illustrated in the system pipeline (Figure 1), the proposed framework operates through a hierarchical data flow. Raw video streams are first pre-processed to reduce noise, and the core logic is then decoupled into two parallel branches: (1) Object Recognition, where YOLO-PEDI extracts bounding boxes; and (2) Spatial Association, where ByteTrack maintains temporal consistency. This integrated design ensures that detection results are accurately ‘pinned’ to specific physical cages via QR-code anchors. When the EAN-13 QR code corresponding to a cage position entered the central region of the field of view, the system automatically triggered a high-frequency snapshot mode to capture key frames. As shown in Figure 1C, because pigeon eggs were frequently partially occluded by the metal mesh during inspection, multi-frame redundant sampling was performed for each cage to improve capture reliability. The data collection process covered a range of practical conditions, including different illumination levels, pigeon occlusion, feather coverage and metal mesh interference. In total, 15,000 raw images were collected. To ensure the high quality and representativeness of the dataset, a rigorous three-stage filtering process was applied to these raw images. First, motion-blur filtering was conducted to remove frames with significant pixel jitter caused by the robot’s movement over rail joints. Second, redundancy reduction was performed to eliminate consecutive frames with high structural similarity to avoid overfitting the model to identical background patterns. Third, target-driven selection was implemented to exclude frames where eggs were 100% occluded by the pigeon’s body or deep nest padding, as these provided no learnable features for object detection. This systematic filtering resulted in a final refined dataset of 4147 high-quality annotated images. For the final experiments, 3015, 566, and 566 images were allocated for training, validation, and testing, respectively.

The test set for broken eggs was set at 50 samples. This relatively smaller sample size compared to normal eggs is due to the inherent rarity of eggshell damage in standardized commercial housing, where the natural breakage rate is strictly controlled. Consequently, the reported metrics for this category should be interpreted as a preliminary robustness indicator within a high-standard production environment.

Due to the inherent scarcity of defective samples in standardized commercial production environments, the test set for abnormal eggs, including broken and deformed eggs, was limited to 50 samples. Consequently, the reported detection metrics for this category should be interpreted as preliminary evidence of model robustness under high-standard housing conditions, rather than as a definitive statistical conclusion. Future longitudinal studies will prioritize the collection of a larger and more diverse anomaly dataset to further validate the robustness and generalizability of the proposed model for abnormal-egg detection.

### 2.2. Design of YOLO-Based Pigeon Egg Inspection Model

**Figure 2:**
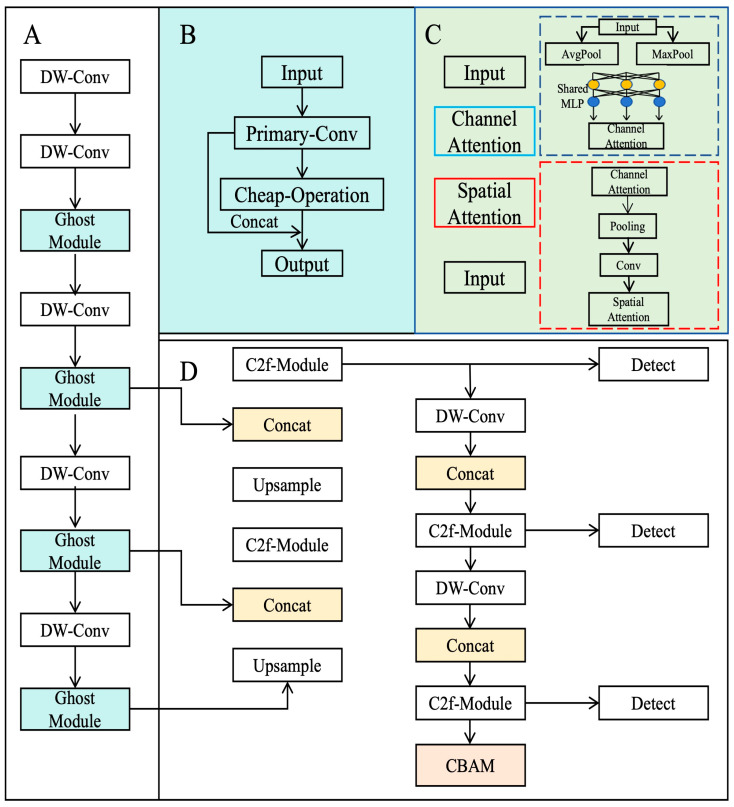
Structure of the YOLO-PEDI network model. (**A**) Lightweight backbone based on stacked Ghost Modules; (**B**) Architecture of the Cheap-Operation module; (**C**) Channel-spatial attention module; (**D**) Neck and detection head incorporating C2f modules and the CBAM attention mechanism.

To address the practical challenges of pigeon egg inspection, including limited computing resources on the robot-mounted edge device, severe background interference, and frequent occlusion by metal cage meshes, an improved lightweight YOLO-PEDI model was developed. The overall architecture is shown in Figure 2. Through lightweight structural redesign and attention enhancement, the proposed model achieves a better balance between detection accuracy and inference speed.

#### 2.2.1. Lightweight Backbone Based on Ghost Modules

As shown in Figure 2A, a lightweight reconstruction of the backbone network was implemented based on the Ghost module [13,17,18]. Standard convolution operations often generate a large number of redundant feature maps, leading to unnecessary computational overhead on embedded platforms. To alleviate this, we introduce the Ghost module to generate feature maps through a two-step process. First, as shown in Figure 2B, a small amount of standard convolution $f_{1\times 1}$ is used to generate essential feature maps $Y^{\prime }$:

(1) $$Y^{\prime }=X\times f_{1\times 1}$$

where $X\in R^{\left(c\times h\times w\right)}$ is the input feature map. Subsequently, additional “Ghost features” are generated using inexpensive linear transformations. According to GhostNet theory, the theoretical speedup ratio $r_{s}$ is calculated as:

(2) $$\;r_{s}=\frac{c\cdot k\cdot k\cdot n}{\frac{n}{s}\cdot k\cdot k\cdot c+\left(s-1\right)\cdot \frac{n}{s}\cdot d\cdot d}\approx s$$

where s represents the number of linear transformations. This design effectively filters redundant background information in the breeding environment while retaining core semantic features, reducing the parameter size to 1.53 M.

#### 2.2.2. Feature Enhancement Mechanism Combined with CBAM

To address the extremely low pixel counts of pigeon eggs caused by metal cage net lines, the YOLO-PEDI algorithm integrates the Convolutional Block Attention Module (CBAM) into the feature fusion network [15,19,23]. As shown in Figure 2C, the module achieves feature recalibration through sequential cascading of Channel Attention and Spatial Attention mechanisms. The channel attention feature calculation is as follows:

(3) $$M_{c}\left(F\right)=\sigma \left(MLP\left(AvgPool\left(F\right)\right)+MLP\left(MaxPool\left(F\right)\right)\right)$$

As shown in Formula (3),where σ represents the Sigmoid activation function. This allows the model to adaptively lock onto specific spectral features of the pigeon egg in chaotic backgrounds. Furthermore, Wise-IoU (WIoU) is introduced in the loss function to improve bounding-box regression accuracy for occluded targets [24]:

(4) $$L_{WIoU}=R_{WIoU}\cdot L_{IoU}$$

The weight hyperparameters for Wise-IoU and the decoupled head were maintained at their standard default values to ensure the model’s generalizability across various poultry farm environments. While these settings were optimized based on empirical evidence from small-target detection tasks, maintaining standard configurations helps mitigate the risk of over-fitting to site-specific lighting or mesh patterns, thereby ensuring stable convergence and robust feature extraction in practical pigeon-cage conditions.

As shown in Figure 2D, the final output layer uses a multi-task decoupled detection head to eliminate interference between localization and classification tasks.

### 2.3. Dynamic Tracking Strategy Based on ByteTrack

**Figure 3:**
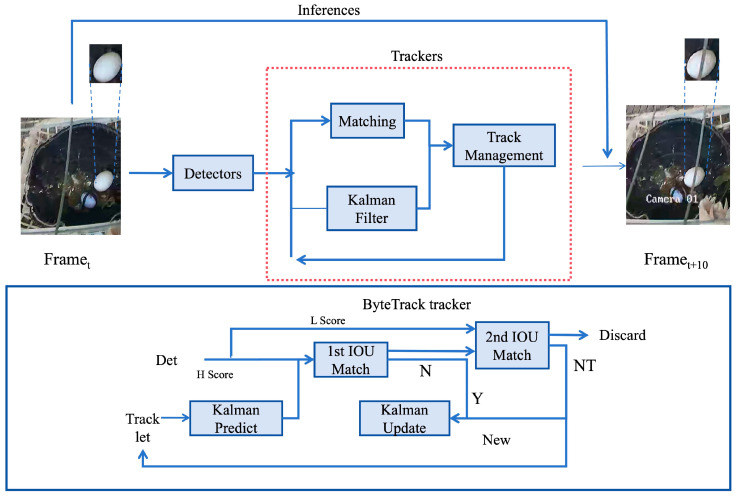
Multi-target tracking system framework and ByteTrack core logic block diagram.

To prevent redundant counting and reliably associate each detected egg with its physical cage location during mobile inspection, we integrated the ByteTrack tracking algorithm [25,26] with QR-code anchors. As shown in Figure 3, the framework consists of a detection input layer, a hierarchical matching–association layer [27], and a spatial anchor logic layer.

#### 2.3.1. Motion Modeling and State Prediction

As shown in the upper part of Figure 3, the tracking process begins with the Detectors extracting features from $Frame_{t}$. To maintain the continuity of pigeon egg identities, the system establishes a motion model based on a Kalman Filter [28,29,30]. The motion state vector $X_{t}$ at moment t is defined as:

(5) $$X_{t}=[x,y,r,h,\dot{x},\dot{y},\dot{r},\dot{h}{]}^{T}$$

where $\left(x,y\right)$ are the center coordinates, r is the aspect ratio, and h is the height. The system predicts the prior state of the current frame based on the posterior estimate of the previous frame, ensuring stable tracking even during temporary occlusions by metal meshes.

#### 2.3.2. Cage-Pigeon Egg Spatio-Temporal Correlation Model

While the tracking algorithm provides continuous trajectory IDs, these identifiers are relative to the video stream and cannot inherently map to absolute physical cage locations. To achieve precise traceability, a spatio-temporal correlation mechanism is established to bind currently tracked pigeon eggs to their respective physical cages. As illustrated in the spatial anchor logic layer of Figure 3, the system treats each cage as an independent operational unit by processing detection and QR-code scanning threads in parallel. During the robot’s movement, a spatial anchoring strategy is employed to resolve the ambiguity of belonging caused by overlapping fields of view between adjacent cages. This is achieved by evaluating the spatial overlap between the pigeon egg detection frame and the predefined Region of Interest (ROI) of the QR code, supplemented by center-distance weighting to ensure identity alignment.

As shown in the core logic block diagram in Figure 3, ByteTrack utilizes a dual-association strategy by dividing detection results into high-score sets $D_{high}$ and low-score sets $D_{low}$:

(6) $$D_{high}=\left\{d_{k}|s_{k}>\tau_{high}\right\},D_{low}=\left\{d_{k}|\tau_{low}<s_{k}\leq \tau_{high}\right\}$$

A mandatory archiving and reset instruction is triggered whenever a new QR code enters the trigger region or the disappearance time of a tracked ID exceeds the threshold. This mechanism ensures that each target is recorded uniquely in the database, effectively preventing redundant counting. Field tests indicate that this spatial anchoring strategy resolves the cumulative ID drift, improving cage attribution accuracy from 85.0% to 99.2%.

As shown in the integrated architecture of Figure 3, the proposed framework consists of three core components: a detection input layer, a hierarchical matching–association layer, and a spatial anchor logic layer. ByteTrack is adopted as the central tracking algorithm, leveraging a two-stage association strategy to ensure identity continuity. In each video frame, detected pigeon eggs are categorized into high-confidence and low-confidence sets based on a predefined threshold. During the 1st stage, high-confidence detections are matched with existing trajectories using the Intersection over Union (IoU) metric. In the 2nd stage, detections with reduced confidence, often caused by metal mesh occlusion or motion blur, are further associated with unmatched trajectories based on positions predicted by the Kalman filter. This hierarchical association strategy effectively reduces identity switches and maintains tracking stability during robot movement. To bridge the gap between virtual trajectories and physical locations, a QR-code-based spatial anchor mechanism is introduced. As illustrated on the right side of Figure 3, the system recognizes cage labels in real-time and dynamically binds tracked target IDs to the corresponding physical cage numbers. When a new QR code enters the predefined trigger region, the previous trajectory–location relationship is decoupled and reassigned according to the current spatial anchor. This closed-loop process establishes a stable mapping among the video stream, tracked IDs, and physical cage numbers, thereby enabling accurate counting, cage-level localization, and traceable management of inspection results.

### 2.4. Dataset Augmentation

This study designed and implemented a multi-dimensional adaptive data augmentation scheme based on the synergy of Mosaic and Mixup in response to practical engineering challenges such as dense small targets, redundant cage and net backgrounds, and intense light fluctuations in large-scale pigeon farms. Based on the original dataset, the Mosaic enhancement strategy reorganizes four unrelated original images into a synthetic sample containing four heterogeneous sub-scenes by randomly scaling, cropping and arranging them. The construction of this spatial heterogeneity geometrically multiplies the target density within a single training batch. For the lightweight model YOLO-PEDI with a parameter size of only 1.53 M, this high-pressure sampling environment forces the Ghost module to capture the core geometry of pigeon eggs within an extremely cramped feature space, significantly enhancing the model’s ability to recognize small-scale targets. Building on this framework, Mix-up augmentation was further introduced to improve model robustness. Mix-up generates cross-semantic training samples by linearly combining two randomly selected images and their corresponding labels. From a regularization perspective, this strategy produces a label-smoothing effect and reduces the tendency of the model to overfit to a single background pattern. By partially decoupling pigeon egg features, such as broken textures and eggshell reflections, from complex non-target backgrounds, including metal mesh and nest padding, Mix-up helps the network learn more generalized feature representations. This process also guides the CBAM to focus on more discriminative spatial regions, thereby improving resistance to background noise under challenging lighting and shadow conditions. Under the high-density augmented training setting, pigeon egg targets exhibit a broader and more discrete scale distribution. This artificially enhanced scale diversity improves the ability of YOLO-PEDI to adapt to viewpoint variation caused by robot motion, without relying heavily on multi-scale testing. In addition, the augmented batch composition improves the model’s ability to distinguish true targets from redundant visual interference, allowing stable detection performance even under strong disturbances such as metallic reflections.

### 2.5. Evaluation Metrics of the Model

The development, training, and ablation tests of the algorithms in this study were all conducted on the same portable computing workstation. The hardware power was provided by 16.0 GB of RAM, an AMD Ryzen 9 7940H processor (4.00 GHz), and an NVIDIA GeForce RTX 4060 graphics card (8 GB of video memory). The software environment runs on the 64-bit Windows 11 operating system, with Python 3.8 as the programming language and the CUDA 11.3 driver integrated with the PyTorch 1.11.0 deep learning framework for accelerated computing.

For the fragmented features of the pigeon egg target, the input resolution for model training is set to 640 × 640 pixels. The optimization algorithm uses stochastic gradient descent (SGD), with an initial learning rate of 0.01. To balance computational overhead and gradient update frequency, set the Batch size to 16 and start 32 parallel worker threads for a total of 50 epochs of iterative training. To enhance the diversity of feature representations and improve model robustness under complex breeding conditions, Mosaic and Mixup data augmentation strategies were employed during the training phase. The Mosaic augmentation combines four training images into one, which effectively introduces a wealth of small-target context and forces the network to identify pigeon eggs across various scales. Complementarily, the Mixup strategy performs convex combinations of image pairs to smooth the decision boundaries, thereby reducing the model’s sensitivity to pixel-level noise and illumination variations. These augmentations simulate the visual complexity of diverse farming environments—such as varying bird densities and cage textures—thereby mitigating over-fitting to the primary data source and strengthening the model’s generalization capacity.

To quantitatively evaluate the recognition accuracy, lightweighting, and real-time performance of the YOLO-PEDI model in the dynamic inspection of pigeon eggs, this paper constructs a multi-dimensional evaluation index system. Recognition performance is mainly evaluated by Precision, Recall, and mean Average Precision; Model complexity and edge-end performance are measured by Parameters, GFLOPs, and Memory usage. The mathematical definitions of each precision metric are as follows:

(7) $$P=\frac{TP}{TP+FP}\times 100\%\;$$

(8) $$R=\frac{TP}{TP+FN}\times 100\%$$

(9) $$AP=\int_{0}^{1}P\left(R\right)dR\;$$

(10) $$mAP=\frac{1}{N}\sum_{i=1}^{N}AP_{i}\;$$

In the above equations, *P* denotes precision, *R* denotes recall, and *mAP* denotes mean average precision. *TP* represents the number of true positive samples, which is positive samples whose predicted results are consistent with the ground-truth labels. *FP* represents the number of false positives, namely negative samples that are incorrectly identified as positive. *FN* denotes the number of false negatives, that is, positive samples that are incorrectly classified as negative. *N* denotes the total number of target instances involved in the classification or evaluation process.

## 3. Results

### 3.1. Training Performance and Ablation Analysis of YOLO-PEDI

We next examined the training dynamics of YOLO-PEDI to assess how the proposed architecture learned to localize and identify pigeon eggs during optimization. To this end, Training loss and mAP@50 were monitored throughout training, as these two metrics capture complementary aspects of model behavior, including bounding-box regression, feature discrimination and convergence stability. Together, they provide a direct readout of convergence speed, parameter efficiency and prediction consistency.

**Figure 4:**
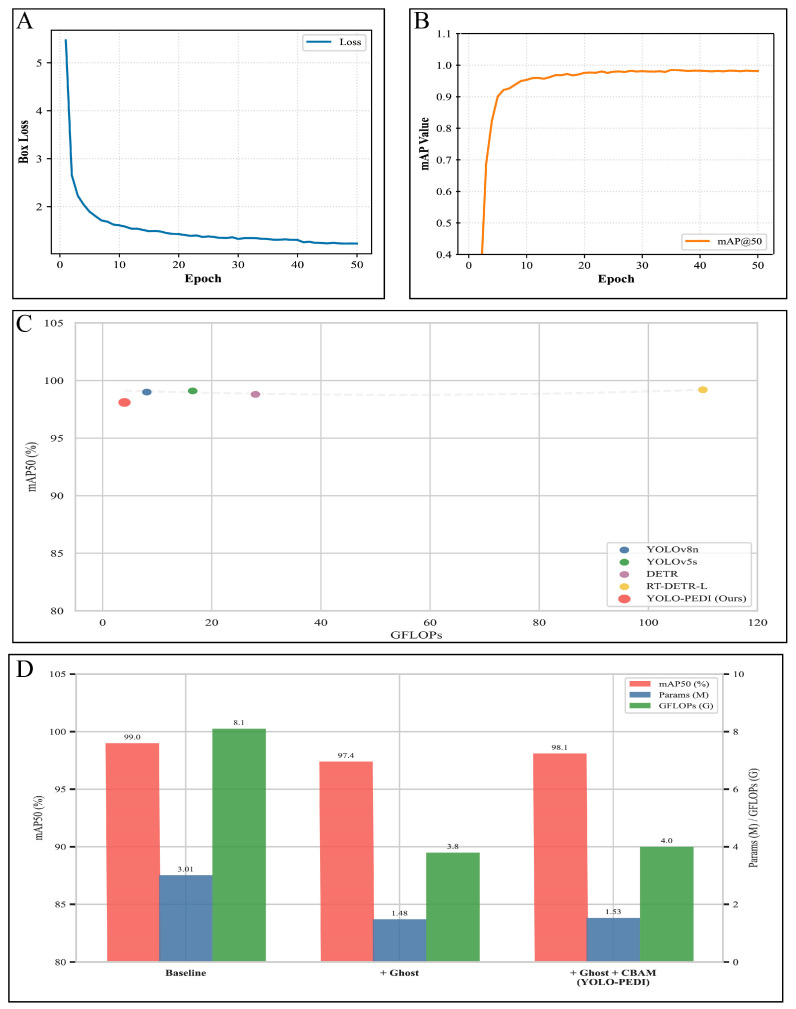
Comprehensive performance analysis of the YOLO-PEDI model. (**A**) Training loss (Box-loss) over 50 epochs; (**B**) mean Average Precision ($mAP_{50}$) curves over 50 epochs; (**C**) Multi-dimensional performance evaluation comparing YOLO-PEDI with other benchmark models; (**D**) Quantified impact of Ghost module and CBAM on model precision and computational complexity in ablation studies.

As shown in Figure 4A, the Training-loss curve showed a rapid decline at the beginning of training, indicating that YOLO-PEDI quickly captured the basic geometric characteristics of pigeon eggs and established an effective localization representation. This early convergence suggests that replacing conventional convolution with Ghost modules preserved the structural cues required for bounding-box regression while reducing redundant computation. As training proceeded, the rate of decline became more gradual and the loss curve entered a stable optimization phase, consistent with progressive refinement of target localization under complex visual conditions. This transition likely reflects the contribution of CBAM, which enhanced the model’s ability to suppress interference from metal cage meshes and feather backgrounds while preserving informative edge features. In the later stage of training, Training loss converged to a low level and remained stable without obvious rebound, indicating that the lightweight architecture retained strong regression capability despite substantial model compression. A similarly favorable pattern was observed for mAP@50. Detection accuracy increased sharply during the early stage of training, reaching 85.0% within the first few training cycles, which points to efficient feature learning from the outset. With continued optimization, mAP@50 increased steadily, exceeded 95.0% as training progressed, and finally converged at 98.1%. The stability of the proposed YOLO-PEDI model is further supported by the smooth convergence of the mAP@50 curve shown in Figure 4B, which reached a steady plateau after approximately 30 epochs, with only minor oscillations of less than 0.5%. This stable convergence behavior indicates that the reported performance was obtained from a well-converged training process on the curated dataset. Nevertheless, repeated training with multiple random seeds and formal confidence-interval estimation would be required in future work to more rigorously quantify the statistical significance of small performance gains.

Notably, the curve remained smooth during the middle and late stages of training, with little evidence of the oscillatory behavior often observed in more heavily parameterized models. This stable high-level convergence indicates that the combination of Ghost-based lightweight feature generation and CBAM-guided feature refinement was sufficient to preserve the diversity and discriminative power of the learned representations, even under complex farm backgrounds. Taken together, these results show that YOLO-PEDI achieved rapid early convergence, stable subsequent optimization and sustained high detection accuracy. The training behavior further suggests that the proposed architectural modifications improved computational efficiency without compromising localization precision or feature discrimination. This combination of efficiency and stability supports the suitability of YOLO-PEDI for real-time pigeon egg inspection in practical farming environments.

To further validate this efficiency-accuracy balance in a broader context, our model was compared against several state-of-the-art architectures. As illustrated in Figure 4C, the positioning of YOLO-PEDI relative to other benchmark models highlights its optimized accuracy-complexity frontier. While the plot appears focused on key representative architectures, these specific data points were selected to bracket the performance limits of both conventional lightweight CNNs and high-capacity transformer-based detectors. This visualization provides the necessary context to demonstrate that YOLO-PEDI achieves a competitive mAP@50 while occupying an ultra-low computational region (4.0 GFLOPs), effectively bridging the gap between the high-precision requirements of egg inspection and the strict latency constraints of mobile robotic platforms.

We next performed ablation experiments to quantify the contribution of each architectural modification and to clarify how lightweight design and attention refinement jointly affected detection performance. Starting from the original YOLOv8n as the baseline, the Ghost module and CBAM were introduced sequentially under a controlled setting and evaluated on the same pigeon egg dataset. This design allowed the effects of parameter reduction and feature enhancement to be assessed independently and in combination, as summarized in Table 1.

The architectural evolution from YOLOv8n to YOLO-PEDI reflects a strategic balance between computational efficiency and feature representation capacity. The intro-duction of Ghost modules is theoretically motivated by the need to reduce redundant feature computations, resulting in a 50.8% decrease in parameter count compared with standard convolutional structures. However, such aggressive lightweight compression may inevitably weaken the representation of fine-grained spatial details, which are critical for detecting small pigeon eggs under cage-mesh interference. To alleviate this limitation, the CBAM block was introduced as an attention-guided semantic refinement module to re-weight feature importance across both channel and spatial dimensions. In this design, Ghost modules mainly provide structural compression, whereas CBAM contributes to feature restoration and target-focused representation. In addition, the loss-design strategy further improves optimization balance by enhancing the model’s robustness to small targets, partial occlusion, and complex backgrounds. This “compression–restoration–balancing” mechanism enables YOLO-PEDI to maintain dis-criminative power while substantially reducing computational cost, thereby achieving a favorable balance between real-time latency and detection accuracy for rail-mounted agricultural inspection robots.

The results reveal a clear functional complementarity between the two modules. Introducing the Ghost module markedly reduced both parameter count and computational cost, with decreases of 50.8% and 53.1%, respectively, relative to the baseline configurations. These reductions confirm the effectiveness of Ghost in replacing redundant convolutional operations with lightweight linear feature generation. This gain in efficiency, however, was accompanied by a modest decrease in mAP@50, indicating that lightweight compression alone led to some loss of discriminative capacity. When CBAM was further incorporated, detection accuracy recovered to 98.1% with only a marginal increase of 0.05 M parameters. This result suggests that the attention mechanism effectively compensated for the representational loss introduced by compression by strengthening informative edge textures and suppressing background interference through joint channel and spatial weighting. The trend shown in Figure 4D further supports this interpretation. Ghost primarily acted as a lightweight structural backbone that removed computational redundancy, whereas CBAM functioned as a feature refinement module that restored fine-grained discrimination. Their combination enabled YOLO-PEDI to maintain high detection accuracy while preserving a compact architecture and fast inference. Together, these findings indicate that the proposed design achieved a favorable balance between accuracy and efficiency, reaching an inference time of 0.8 ms without sacrificing detection reliability.

### 3.2. The Performance of Different Base Detection Algorithms

To rigorously evaluate the competitiveness of YOLO-PEDI for large-scale pigeon farm inspection, we performed a cross-architecture comparison using representative detectors from both conventional and emerging paradigms. The comparison included Faster R-CNN with a ResNet-50 backbone as a two-stage benchmark, YOLOv5s and YOLOv8n as widely used one-stage industrial baselines, and DETR and RT-DETR-L as transformer-based detectors with global modeling capability. All models were trained and tested under the same dataset distribution and hardware conditions (NVIDIA RTX 4060 GPU with 8 GB VRAM), and the quantitative results are summarized in Table 2.

The comparative results reveal clear differences in the trade-off between accuracy and efficiency across architectures. Faster R-CNN retained the classical advantage of two-stage detectors in feature refinement but showed a pronounced efficiency bottleneck in dense pigeon egg detection. Its region-proposal-based design resulted in a computational cost of 180.2 GFLOPs and an inference time of 85 ms, which is difficult to accommodate in dynamic inspection scenarios. Under mobile robot operation, such latency can lead to delayed response, repeated counting, and increased risk of missed detections. By contrast, YOLO-PEDI adopted a one-stage end-to-end detection framework and substantially reduced inference time to 0.8 ms through lightweight architectural redesign, demonstrating a clear advantage for edge-side deployment in resource-constrained inspection systems.

Transformer-based detectors, particularly RT-DETR-L, achieved the highest accuracy among the compared models, reaching a mAP@50 of 99.2%. This result reflects the strength of global self-attention in capturing targets under dense and cluttered backgrounds. However, this gain in accuracy came at the expense of substantially higher model complexity and memory demand, with a parameter size of 32.0 M. Rather than pursuing the highest single-metric performance, YOLO-PEDI was designed to achieve a more balanced solution for practical deployment. By introducing CBAM, the model compensated for the limited global modeling ability of conventional convolutional networks through channel and spatial attention. As a result, YOLO-PEDI maintained a mAP@50 of 98.1% while using only a small fraction of the parameters required by RT-DETR-L, indicating that attention-guided lightweight CNNs remain highly competitive for this task.

A key strength of YOLO-PEDI lies in its efficient handling of feature redundancy. Conventional convolutional layers often generate highly similar feature responses, leading to unnecessary computational overhead. By replacing part of this redundant computation with the Ghost module, YOLO-PEDI generated representative intrinsic features using a reduced number of standard convolutions and then produced additional feature maps through inexpensive linear operations. This design reduced the computational load to 4.0 GFLOPs, which was only 49.3% of that of YOLOv8n. Although a slight decrease in mAP@50-95 was observed under this aggressive compression, the reduction was limited relative to the substantial savings in computational resources. Given that pigeon egg inspection primarily requires reliable counting and status recognition rather than extremely strict localization precision, this trade-off is acceptable and practically meaningful for smart farming applications.

The overall comparison further confirms this conclusion. As shown in Figure 4C, YOLO-PEDI remained comparable to mainstream benchmark models in detection accuracy, while showing clear advantages in model lightweighting and inference efficiency. This asymmetric performance profile is particularly valuable in real deployment scenarios, where hardware cost, power consumption and real-time response are all critical constraints. The compact design of YOLO-PEDI makes it suitable for deployment on low-cost embedded platforms, while its high processing speed can increase inspection coverage within a fixed patrol cycle. Taken together, these results show that YOLO-PEDI achieves a favorable balance between accuracy and speed in automatic pigeon egg detection and provides an effective technical solution for edge-side vision systems in future large-scale smart farms.

Furthermore, to address the limitation of benchmarking on a desktop GPU, the YOLO-PEDI model was exported to ONNX format for cross-platform profiling. The exported model has a compact footprint of 3.1 MB and a computational complexity of 4.0 GFLOPs, corresponding to only 49.3% of the computational cost of the baseline YOLOv8n. These hardware-agnostic metrics indicate that the proposed architecture is substantially lightweight and structurally suitable for resource-constrained deployment scenarios. However, the reported 0.8 ms inference time should be interpreted as the latency measured on the RTX 4060 desktop GPU used in this study, rather than as the actual latency on embedded edge devices. Direct profiling on target platforms such as Jetson Nano, Jetson Orin Nano, Raspberry Pi, or the final robot controller remains necessary to fully quantify real-world edge-device performance. Future work will therefore include embedded-platform benchmarking under practical robot operating conditions to more rigorously evaluate deployment efficiency.

### 3.3. Grad-CAM-Based Visualization of Pigeon Egg Localization

**Figure 5:**
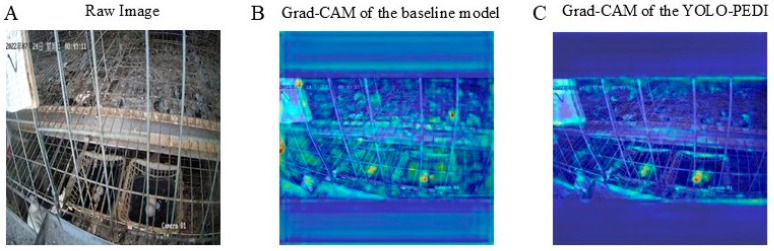
Comparative visualization of model interpretability based on Grad-CAM. (**A**) Raw image of the pigeon house environment. (**B**) Heatmap of the baseline model, showing diffused activation patterns and significant response overlap with the metal cage mesh. (**C**) Heatmap of the improved YOLO-PEDI, demonstrating a precise focus on the pigeon egg contours while effectively suppressing background interference from the mesh and feathers.

We employed Grad-CAM to visualize the feature activation patterns of YOLO-PEDI and to assess its ability to focus on target regions under complex breeding conditions. Representative activation maps are shown in Figure 5. In panoramic pigeon-house images, eggs occupy only a small portion of the field of view and are frequently affected by cage-mesh interference, background clutter, and illumination variation. Under these challenging conditions, YOLO-PEDI generated compact and spatially concentrated activation maps. The strongest responses were mainly aligned with the central regions and elliptical contours of the eggs, indicating that the model captured local features that are relevant for precise detection.

To further examine the interpretability gained from the architectural refinements, we conducted a comparative Grad-CAM analysis between the baseline model and YOLO-PEDI. As shown in Figure 5, the baseline model produced relatively diffused activation patterns, with noticeable responses on non-target structural elements such as repetitive metal-mesh lines, cage edges, and background padding. This suggests that the baseline network was more susceptible to feature distraction caused by high-frequency farm artifacts.

After the integration of Ghost modules and CBAM, the activation pattern showed a clear transition from diffused background response to more localized target-focused attention. The high-response regions became more concentrated around the biological contours of pigeon eggs, while responses from shaded areas, metal cage edges, and other non-target regions were reduced. This comparison provides qualitative evidence that the attention-refinement mechanism helps suppress spurious background activations and improves the model’s focus on target-relevant features. Nevertheless, the present Grad-CAM analysis remains qualitative, and quantitative evaluation of activation overlap with ground-truth masks was not conducted in this study. Future work will introduce quantitative interpretability metrics, such as activation–mask overlap ratios or pointing-game analysis, to further evaluate the localization consistency of model attention.

### 3.4. ByteTrack-Based Continuous Counting of Pigeon Eggs

Although ByteTrack effectively handles short-term occlusion during continuous inspection, its trajectory memory can also introduce systematic errors in high-density cage environments. During long patrols, motion-prediction inertia may cause target IDs from one cage to drift into adjacent cages, leading to cumulative counting errors across cages. To overcome this limitation, we introduced a QR-code-forced threshold mechanism as a spatial anchor for trajectory reset and cage-level isolation.

The mechanism uses a front-end visual unit mounted on the inspection robot to identify cage QR codes in real time. Once a new cage code enters the predefined recognition region, all cached trajectories in the current ByteTrack tracker are immediately cleared and the counting results of the current cage are archived. In this way, each cage is treated as an independent spatiotemporal unit, thereby preventing cross-cage error accumulation. Quantitative evaluation showed that, before this mechanism was introduced, target misassociation produced an average cumulative counting error of approximately 15.0% per 50 cages. After QR-code-based forced thresholding was applied, cage attribution accuracy increased from 85.0% to 99.2%. These results indicate that the proposed strategy effectively eliminated counting bias caused by long-range trajectory drift and provided a reliable basis for cage-level digital management under a “one cage, one record” framework.

**Figure 6:**
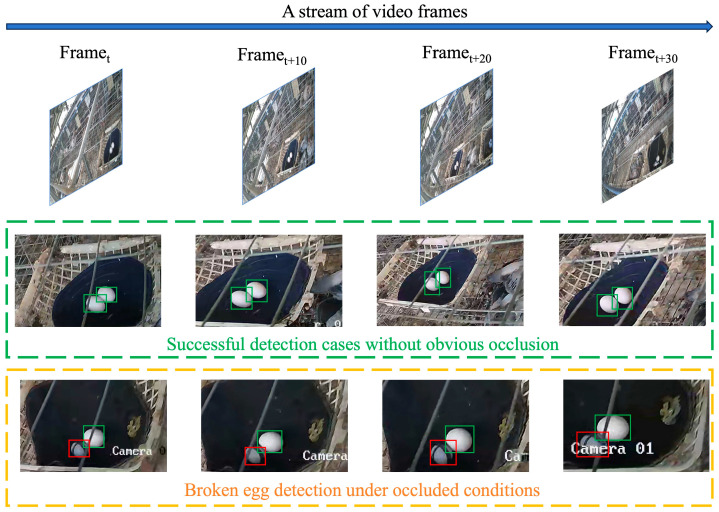
Actual test results of the track-based pigeon egg intelligent inspection system.

To further evaluate system performance under practical farming conditions, a 30 min inspection video was collected from a commercial pigeon house. During the test, the inspection robot moved at a constant speed while the onboard camera continuously captured cage images, and the video stream was processed online using the improved YOLO-PEDI model. Representative detection results are shown in Figure 6. The system maintained stable performance under complex backgrounds and partial occlusion. As shown in Figure 6, during robot movement, the optical axis frequently intersected the metal cage mesh at different angles, resulting in strong visual interference. Even when pigeon eggs were partially blocked by thick galvanized wires or occluded by the pigeon body, YOLO-PEDI remained able to localize the target accurately. This robustness is consistent with the enhanced feature selection enabled by the attention mechanism, which allowed the model to preserve informative texture and contour cues under challenging visual conditions. The resulting stable detections also provided reliable input for subsequent ByteTrack-based counting and association. In addition to counting, the system simultaneously performed egg-quality recognition. As shown in Figure 6, the model successfully distinguished normal pigeon eggs from broken eggs in dynamic inspection scenes. For the key production indicator of broken eggs, the system achieved a recall of 98.0%, indicating high sensitivity to fine defect features. This result suggests that the proposed model retained sufficient discriminative capacity to identify subtle structural abnormalities, such as cracks and surface damage, despite its lightweight design.

The overall results of dynamic inspection are summarized in Table 3. The system showed particularly strong performance in detecting high-value production indicators, especially broken eggs. Although dense occlusion occasionally caused trajectory interruption and affected cumulative counting accuracy, the overall counting accuracy still reached 80.9%. By introducing the QR-code-forced threshold mechanism, the system established a closed-loop association among video frames, cage numbers and detected targets. In field tests, with the support of supplemental illumination, the QR-code recognition rate remained above 99.5%, ensuring that each detected broken egg could be accurately traced back to its corresponding physical cage. This cage-level traceability mechanism effectively resolves a common limitation of traditional inspection, in which broken eggs can be detected but cannot be reliably assigned to a specific cage.

To complement the overall inspection results, we further quantified system performance in terms of tracking stability and processing speed using 15 inspection videos collected under different time periods and illumination conditions, comprising approximately 36,000 frames in total. The multi-object tracking accuracy (MOTA) remained stable at 88.6% when the patrol robot moved at a constant speed of 0.5–0.8 m/s. The variance of MOTA fluctuation remained below 0.04, even under frequent image jitter caused by rail joints, indicating strong tracking robustness during continuous motion. This stability is consistent with the secondary association mechanism in ByteTrack, which links low-confidence detections to historical trajectories and thereby reduces track fragmentation under sudden changes in illumination and partial occlusion.

The system also maintained favorable ID consistency and real-time performance. During a complete inspection trip, the average number of identity switches was only 1.2 per 1000 frames, indicating strong trajectory continuity and reliable counting uniqueness. In terms of computational efficiency, the average processing speed reached 68.4 FPS, with a peak speed of 82 FPS. This throughput exceeds the 30 FPS acquisition rate of the industrial camera and provides sufficient computational margin for additional edge-side functions, such as multi-sensor fusion and real-time warning. Together, these results demonstrate that the proposed system can deliver stable, traceable and real-time inspection performance in practical smart pigeon farming environments.

#### 3.4.1. Analysis of Typical Failure Modes

**Figure 7:**
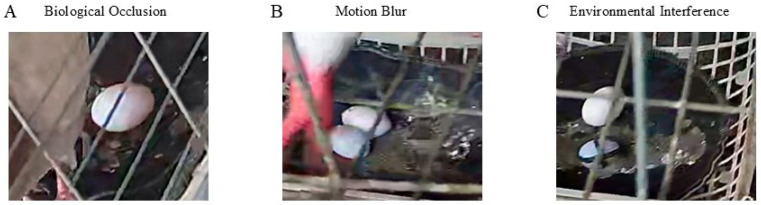
Analysis of Representative Failure Cases.

To provide a more explicit understanding of the gap between the model-level mAP and the reported 80.9% cumulative counting accuracy, we conducted a systematic review of the recognition logs and representative video frames obtained during the field experiment. As illustrated in Figure 7, the three major failure cases were identified: biological occlusion, motion blur, and environmental interference.

Biological occlusion was the dominant factor, accounting for approximately 70% of missed detections. In these scenarios, breeding pigeons physically covered the eggs to provide warmth or protection, causing the eggs to become completely invisible to the overhead camera. Therefore, even a highly accurate detection model cannot identify these targets when no visual evidence is available in the input image. Motion blur was another important contributor to counting errors. When the robot operated at speeds approaching 0.8 m/s, rail vibration and platform motion occasionally caused image jitter or blurred egg boundaries. This could destabilize the Kalman-filter-based prediction and data association process in ByteTrack, resulting in identity fragmentation, temporary target loss, or duplicate counts. Environmental interference also affected counting stability under certain viewing angles. Structural elements such as feeding troughs, thick galvanized wires, and nest padding created localized blind spots and partially obscured the elliptical contours of the eggs. These factors increased the difficulty of feature extraction in highly cluttered cage environments. The integration of the QR-code-based “hard reset” mechanism mitigated the impact of these failure events at the system level. By periodically reinitializing the spatial association at each cage unit, tracking interruptions were localized within individual cages, preventing error accumulation and propagation across adjacent cage units. This mechanism helped preserve the overall integrity of the production database under practical farm conditions.

#### 3.4.2. System Robustness Under Challenging Field Conditions

The operational reliability of the YOLO-PEDI-based inspection framework was further analyzed under several challenging field conditions commonly encountered in commercial pigeon houses, including illumination heterogeneity, increased robot speed, and spatial-anchor degradation.

In multi-tier cage structures, illumination can vary considerably across different cage levels, with lower-tier cages often suffering from insufficient ambient light. To address this issue, the patrol robot was equipped with integrated LED arrays to provide supplementary illumination and maintain an adequate signal-to-noise ratio in poorly lit areas. In addition, the CBAM-enhanced backbone can strengthen the model’s attention to stable structural features, such as egg contours, while suppressing interference from low-contrast background textures. This design is expected to improve detection stability under heterogeneous illumination conditions.

Robot speed is another important factor affecting image quality and tracking continuity. During routine inspection, the robot operates at approximately 0.2 m/s, at which no obvious motion blur is typically observed. When the speed approaches 0.8 m/s, however, rail-induced vibration and platform motion may introduce motion blur or image jitter into the video stream. Under such conditions, ByteTrack’s Kalman-filter-based motion prediction can help maintain short-term trajectory continuity by estimating target locations from historical motion states, thereby reducing temporary target loss and identity switches. Nevertheless, excessively high speeds may still degrade image sharpness and counting stability, indicating that operational speed should be controlled within a reasonable range for reliable deployment.

The robustness of the spatial-anchor chain was also considered for partially occluded or degraded QR codes. In practical farm environments, QR labels may become partially unreadable because of dust, feathers, manure contamination, physical wear, or viewing-angle changes. To handle this situation, the system adopts a “graceful degradation” strategy. If a cage-level QR code cannot be decoded, the system temporarily relies on short-term visual tracking and maintains the current spatial association until the next valid QR code is detected. Once a clean QR code is recognized, the spatial mapping is reinitialized through the QR-code-based “hard reset” mechanism. This strategy localizes tracking interruptions within individual cage units and prevents identity drift or counting errors from propagating across the entire inspection row.

## 4. Discussion

### 4.1. Analysis of Failure Modes and System Robustness

Regarding the 80.9% cumulative counting accuracy, we identified three primary failure modes through detailed video review. First, biological occlusion is the most significant factor, accounting for approximately 70% of missed detections; breeding pigeons often exhibit protective behavior, completely covering eggs with their bodies, which leads to target disappearance in the visual stream. Second, motion-induced instability caused by rail vibrations at higher robot speeds (up to 0.8 m/s) occasionally destabilizes the Kalman filter predictions in ByteTrack, resulting in identity fragmentation. Third, environmental interference from feeding troughs and nest padding creates localized blind spots. Potential failure modes in the spatial anchor chain, such as QR-code occlusion, motion blur, or lighting variation, were further evaluated. In scenarios where QR codes are partially occluded or degraded by environmental stains, the system maintains short-term tracking continuity via ByteTrack’s Kalman Filter predictions. While prolonged QR-code failure might eventually lead to identity drift, the periodic appearance of subsequent clean labels ensures that counting errors remain localized within a specific cage segment, preventing the propagation of drift across the entire inspection row. To address these limitations, the integration of QR codes as cage-level spatial anchors provides a “hard reset” mechanism. By transforming each cage into an independent spatiotemporal unit, the system prevents error propagation across adjacent cages, which substantially improved cage attribution accuracy from 85.0% to 99.2%. This design offers a more robust solution for structured farm environments compared to fully vision-based localization. Even under tracking failure, the spatial anchor chain ensures that the “one cage, one record” paradigm remains intact.

### 4.2. Architectural Synergy and Practical Value

A key advance of this work is the development of YOLO-PEDI, which preserves high detection performance within a highly compact architecture. The Ghost module reduced computational redundancy, whereas CBAM selectively enhanced informative spatial and channel responses. Beyond the incremental combination of existing modules, the core contribution of YOLO-PEDI lies in its domain-specific engineering optimization tailored to the practical constraints of agricultural edge robotics. Unlike general-purpose detectors designed primarily for benchmark performance, YOLO-PEDI follows a constraint-driven design philosophy. In this framework, the Ghost module and CBAM are not simply appended as independent plug-ins but are strategically integrated to address the fundamental conflict between extreme lightweight requirements and reliable feature representation in visually cluttered poultry-house environments. The Ghost module reduces redundant feature computation and enables a sub-1.6 M parameter model suitable for low-power robotic deployment, whereas CBAM enhances attention to target-relevant egg contours under high-frequency interference from galvanized cage meshes and other structural backgrounds. This targeted compression–refinement strategy provides a practical engineering solution for resource-constrained smart farming, where computational efficiency is a prerequisite for transferring high-accuracy detection from workstation-based experiments to stable, battery-powered field operation on mobile inspection robots. This synergy explains why YOLO-PEDI achieved a favorable balance between an mAP50 of 98.1% and an inference speed of 0.8 ms, making it well-suited for edge-side deployment on mobile robots.

### 4.3. Limitations and Future Directions

The practical value of the system lies in its ability to link each recognition event to a specific cage in real time. While the practical value of the proposed system was validated through field deployment, we acknowledge that the custom dataset used in this study was collected from a single commercial pigeon farm and is currently non-public. These factors limit direct independent verification and may affect the external validity of the reported results across farms with different cage structures, lighting configurations, bird densities, management practices, and camera installation settings. To the best of our knowledge, there is currently no publicly available benchmark dataset specifically designed for caged pigeon-egg detection under rail-mounted robotic inspection conditions. Therefore, a direct quantitative comparison with existing public datasets may not provide a strictly equivalent assessment of domain shift because of differences in target category, imaging perspective, occlusion pattern, background structure, and inspection protocol.

To partially mitigate the risk of environment-specific overfitting, we adopted data augmentation strategies, including Mosaic and Mixup, to simulate visual variations caused by illumination changes, object-density differences, occlusion, and complex cage backgrounds. In addition, we provided a detailed description of the data curation and annotation process in Section 2.1 to improve methodological transparency. Nevertheless, cross-farm validation and benchmark comparison remain important limitations of the present study. Future work will prioritize multi-site data collection, public release of anonymized datasets when permitted by farm privacy and biosecurity requirements, and quantitative domain-shift evaluation using suitable poultry-egg or small-object detection benchmarks. Validating the system’s performance across heterogeneous housing structures remains a primary objective for future research. Under extreme conditions such as severe occlusion or very low illumination, the recall for broken-egg detection may still decline. Future work should explore multi-view fusion strategies to mitigate visual blind spots and investigate the integration of multimodal sensing, such as thermal or infrared imaging. Although the current implementation adopts empirically validated default settings for the loss functions and detection heads, a comprehensive sensitivity analysis of the weighting hyperparameters was not conducted in the present study. Therefore, the robustness of the multi-task decoupled head and Wise-IoU loss under different weighting configurations remains to be further quantified. Future research will focus on evaluating the impact of hyperparameter variations on detection accuracy, convergence stability, and counting reliability under more diverse field conditions, including different cage densities, illumination spectra, occlusion levels, and abnormal-egg sample distributions.

Regarding system scalability and operational efficiency, the proposed framework shows potential for large-scale deployment involving dense cage arrays. The end-to-end throughput of 68.4 FPS, measured on the test workstation, indicates that the framework has a substantial computational margin. However, this result should be interpreted as workstation-based evidence of processing efficiency rather than a direct measurement of embedded-platform throughput. For practical deployment, further profiling on the final edge controller remains necessary.

The lightweight design of YOLO-PEDI, with a compact model footprint of 3.1 MB, is expected to reduce computational load and memory-access demand, which are important prerequisites for battery-powered inspection robots. Nevertheless, direct battery-level power consumption was not measured in this study, and future work will include embedded-platform energy profiling under continuous robot operation.

In scenarios where tracking fails due to prolonged heavy occlusion, the system follows a graceful degradation strategy through its spatial-anchor mechanism. When eggs remain invisible because of biological occlusion, temporary missed counts or cage-level uncertainty may occur. However, the QR-code-based hard reset mechanism prevents such errors from accumulating across the entire inspection row. Once a valid spatial anchor is detected, the cage association is reinitialized, thereby localizing tracking failures to individual cage units and preserving the overall integrity of the “one cage, one record” production database. Future research will further evaluate the system across different housing structures and larger-scale farms, while also exploring multi-view fusion and multimodal sensing, such as infrared imaging, to mitigate visual blind spots caused by biological occlusion.

## 5. Conclusions

This study developed an intelligent inspection and localization system that integrates the lightweight YOLO-PEDI detector with QR-code-assisted dynamic tracking to address two major challenges in large-scale pigeon farming: the low efficiency of manual inspection and the weak association between visual recognition results and physical cage locations. By combining lightweight visual perception, continuous target tracking and cage-level spatial anchoring, the proposed framework extends conventional object detection into a practical solution for real-time inspection in structured breeding environments.

A central contribution of this work is the design of the YOLO-PEDI model. By introducing the Ghost module to replace part of the standard convolution operations, the model substantially reduced parameter size and computational cost to 1.53 M parameters and 4.0 GFLOPs. Compared with the baseline YOLOv8n, this design achieved nearly 50% model compression while retaining strong feature extraction capability. With an inference time of only 0.8 ms per frame, the model markedly reduced dependence on onboard computing resources and provided the basis for real-time deployment on mobile inspection robots. To compensate for the potential loss of discriminative capacity caused by aggressive light-weighting, CBAM was further incorporated to enhance attention to subtle yet informative features, including eggshell cracks and deformed contours, while suppressing interference from metal meshes, bedding textures and other background noise. As a result, the model maintained a mAP@50 of 98.1% under complex farming conditions, demonstrating that lightweight design and high detection accuracy can be achieved simultaneously in this task. Another key contribution is the introduction of a ByteTrack-based continuous counting strategy with QR-code spatial anchoring. By combining trajectory association with cage-level QR-code resetting, the system effectively reduced identity drift, repeated counting, and cross-cage error accumulation during long patrols. The QR-code-forced threshold mechanism improved cage attribution accuracy from 85.0% to 99.2%, while the system maintained a real-time processing speed of 68 FPS. For broken pigeon eggs, which represent a critical production indicator, recall reached 98.0%. These results enabled a stable closed-loop mapping among video streams, target trajectories and physical cage identities, thereby supporting the one cage and one record management paradigm.

Overall, the proposed system achieved a favorable balance among detection accuracy, computational efficiency, and deployment practicality. It provides an effective technical solution for real-time intelligent inspection in large-scale pigeon farms and offers a useful reference for edge-side vision systems in other livestock and poultry production scenarios.

## Figures and Tables

**Table 1:** Summary Comparison of ablation Experiment Results of the YOLO-PEDI model.

Protocol	Basic Model	Ghost Module	CBAM Attention	mAP50 (%)	Params (M)	GFLOPs (G)	Latency (ms)
1	√			99.0	3.01	8.1	1.0
2	√	√		97.4	1.48	3.8	0.7
3	√	√	√	98.1	1.53	4.0	0.8

**Table 2:** Performance comparison of different object detection algorithms on the pigeon egg dataset.

Model Algorithm	Architecture Type	mAP50 (%)	mAP50–95 (%)	Params (M)	GFLOPs (G)	Latency (ms)
Faster R-CNN	Two-stage	96.5	58.2	41.20	180.2	85.0
YOLOv5s	One-stage	99.1	71.8	7.20	16.5	1.5
YOLOv8n	One-stage	99.0	67.3	3.01	8.1	1.0
DETR	Transformer	98.8	69.9	41.00	28.0	25.0
RT-DETR-L	RT-Trans.	99.2	72.5	32.00	110.0	12.0
YOLO-PEDI	One-stage	98.1	65.4	1.53	4.0	0.8

**Table 3:** Performance of classification recognition and cumulative counting in dynamic inspection.

Detection of Categories	True Quantity/Piece	Detected Quantity/Pieces	Missed Detections/Pieces	Recognition Accuracy/%
Normal pigeon eggs	1000	800	200	80
Broken pigeon eggs	50	49	1	98
Overall indicators	1050	849	201	80.9

## Data Availability

The data presented in this study are available on request from the corresponding author.
